# Recent and future grand challenges in protein folding, misfolding, and degradation

**DOI:** 10.3389/fmolb.2014.00001

**Published:** 2014-03-27

**Authors:** Pierre Goloubinoff

**Affiliations:** DBMV, Faculty of Biology and Medicine, University of LausanneLausanne, Switzerland

**Keywords:** aggregation, proteopathies, crowding, molecular chaperones, proteases

Our general aim is to better understand the biophysical and biochemical principles that govern nascent or stress-destabilized proteins to unfold and re/fold to their native state or, alternatively, choose to misfold and aggregate into cytotoxic species, which in animals can cause degenerative diseases.

When a protein is first artificially unfolded by a chaotropic agent, it may thereafter spontaneously refold into its three dimensional native state without necessitating additional steric information from other macromolecules in the solution. Anfinsen (1973), thus masterfully demonstrated that the primary sequence of the amino acids contains sufficient information for polypeptides to reach their native functional three-dimensional structure. Yet, the yields of such *in vitro* protein refolding assays are typically low and a large fraction of the polypeptide precipitates into insoluble aggregates.

Living cells cannot accumulate high concentrations of chaotropic agents to specifically unfold potentially toxic aggregates, while leaving intact the surrounding native proteins. Instead they express molecular chaperones that can specifically recognize and bind with high-affinity to the misfolded polypeptides, which are to be unfolded into intermediates that can, thereafter, spontaneously refold into low-affinity native products (Hinault et al., 2006; Priya et al., 2013). In the cell, heat-stress can cause the partial transient opening of native proteins, typically at hinges between compact domains, and partially deconvoluted segments may then seek default stable conformers enriched with beta-sheet structures and with hydrophobic surfaces partially exposed to the aqueous phase (Natalello et al., 2013). Such intermediate species may then seek to reduce undesirable interactions with water and gain stability by forming strong cooperative hydrophobic hydrogen-bonds with other encountered intermediates, in a concentration-dependent oligomerization process generally dubbed “aggregation.” The propensity of early-misfolded polypeptide species to form large insoluble aggregates depends on their initial concentration Dobson (2004) and also on the presence of small pre-formed aggregates that can act as seeds to the aggregation reaction (Gidalevitz et al., 2006).

In the last two decades, a major discovery has been that misfolded and aggregated conformers, even from “normal” non-toxic proteins, can become cytotoxic and infectious (Ben-Zvi and Goloubinoff, 2002). The proteotoxic effects of various pre-amyloid and proto-fibrillar forms of misfolded and aggregated proteins is a hallmark for many degenerative protein conformational diseases and is thought to be the main cause for late onset proteinopathies, such as Parkinson's, Alzheimer's, Amytrophic lateral sclerosis and diabetes types 2 (Luheshi and Dobson, 2009).

It is not clear why some early forms of aggregated proteins are toxic to mammalian cells, especially to neurons, where they apparently induce apoptosis. It is suggested that protofibrils may spontaneously form holes in membranes and disturb biological membranes in general (Lashuel and Lansbury, 2006). Moreover, they can lure other metastable proteins to misfold, aggregate, and become toxic, instead of spontaneously reverting after stress into harmless native proteins. Moreover, misfolded protein intermediates may stall the network of molecular chaperones and proteases that otherwise would effectively act as defenses of animal cells against proteotoxic conformers.

Even without aging and abiotic stresses, when polypeptides are synthetized in the ribosome, growing chains encounter the highly crowded environment of the cytoplasm, which is composed of up to 200 mg/ml proteins (Finka and Goloubinoff, 2013) and of ion and organic molecules in the hundreds millimolars range (Diamant et al., 2001). Cellular crowding is generally thought to favor misfolding and aggregation of unfolded polypeptides, such as new polypeptides emerging from the ribosome (Becker et al., 2013) or from an import pore. Yet, the presence of high concentrations of osmolytes or native proteins may stabilize already native proteins (Melo et al., 2010).

**Figure 1:**
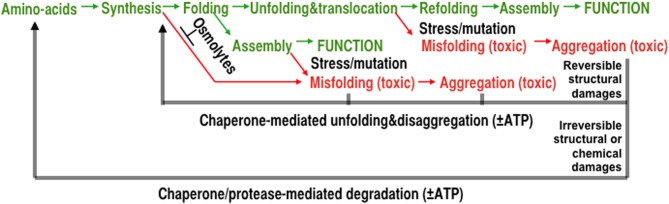
**The various mechanisms of protein homeostasis**. The maturation process of a polypeptide, from synthesis to the assembly of a native functional oligomer (green) is composed from delicate successive processes that can be affected by mutations, chemical modifications, and stress. Under stressful conditions, polypeptides may convert into misfolded and increasingly insoluble aggregates lacking specific biological activities. Misfolded conformers can also induce misfolding of other metastable polypeptides, damage membranes, and in animal cells, cause apoptosis and tissue loss, aging and degenerative diseases. Whereas molecular chaperones can prevent and actively repair structural damages in reversibly damaged aggregates, proteases can degrade and recycle irreversibly damaged protein conformers.

While emerging from the ribosome, intrinsically unfolded polypeptides may stay unfolded and, depending on the presence of osmolytes, pre-aggregated seeds or of stressful conditions, they may misfold and aggregate into toxic species as in the cases of α-synuclein or tau (Luheshi and Dobson, 2009). Other *de novo* synthesized polypeptides may readily fold to the native state, as initially shown *in vitro* by Anfinsen in the simple case a monomeric enzyme. At variance, the concomitant or sequential assembly of multineric complexes may necessitate molecular chaperones to maintain near-native subunits in an assembly-competent state until other subunits are present to form functional oligomers. Polypeptides may also have to be prevented by cytoplasmic chaperones from completing folding while still in the wrong compartment and thus be maintained in a translocation-competent state poised to translocate through membrane pores to the stroma of mitochondria, chloroplasts or the lumen of the *endoplasmic reticulum* (Figure 1. green paths).

Depending on the severity of various abiotic stresses, such as heat-shock, or owing to destabilizing chemical modifications or mutations, polypeptides may misfold and aggregate into potentially toxic conformers (Figure 1, red paths). Whereas osmolytes and so-called “holding” chaperones may prevent misfolding and aggregation, other chaperones can use the energy of ATP-hydrolysis to forcefully unfold and dissolve stable protein aggregates. Even without ATP hydrolysis, mere binding to particular molecular chaperones can unfold loosely misfolded species, which upon release, can refold to the native state. Thus, unfolding chaperones may provide renewed chances to already misfolded polypeptides to spontaneously refold into native functional proteins (Figure 1, black middle arrow). In case the aggregated conformers become irreversibly damaged structurally or chemically, chaperone-gated proteases can use the energy of ATP to forcefully unfold and solubilize aggregates into hydrolysable polypeptides, thereby generating free amino acids to re-synthetize new polypeptides (Figure 1, black lower arrow).

*Frontiers in Molecular Biosciences, Protein folding, Misfolding, and Degradation* publishes original biochemical research article addressing the processes of protein synthesis, folding, misfolding, translocation, refolding, assembly, and aggregation, as well as specific mechanisms by which chemical chaperones, molecular chaperones and proteases control cellular protein homeostasis. Of particular interest are physiological cellular processes, such as *de novo* folding and assembly of native complexes, which do not necessarily involve chaperones preventing or reverting protein aggregation. How may chaperones differentiate bound on-pathway near-native subunits seeking to integrate native oligomers, from off-pathway oligomer-orphan subunits that become rapidly degraded?

We are primarily interested in biochemical and molecular mechanistic studies addressing how chaperones and proteases use ATP hydrolysis to unfold, disaggregate and promote the native folding or the degradation of stably misfolded and aggregated proteins species and about the principles governing partitioning between the “rehabilitation” of misfolded proteins by unfolding and disaggregating chaperones, and “recycling” of misfolded proteins by unfolding proteases (Suraweera et al., 2012). We are also interested in proteomic biochemical and structural studies addressing the ways by which various misfolded and aggregated proteins accumulate in cells, and in the *in vivo* imaging of protein misfolding and amyloid accumulation in degenerative tissues and whole organisms.
